# Public Knowledge and Attitudes towards Vitiligo: A Survey in Mekelle City, Northern Ethiopia

**DOI:** 10.1155/2020/3495165

**Published:** 2020-05-31

**Authors:** Afewerki Gebremeskel Tsadik, Mezgebe Zeru Teklemedhin, Tesfay Mehari Atey, Meles Tekie Gidey, Desilu Mahari Desta

**Affiliations:** ^1^Clinical Pharmacy Course and Research Unit, School of Pharmacy, College of Health Sciences, Mekelle University, Mekelle, Tigray, Ethiopia; ^2^Pharmacoepidemiology and Social Pharmacy Course and Research Unit, School of Pharmacy, College of Health Sciences, Mekelle University, Mekelle, Tigray, Ethiopia

## Abstract

**Background:**

The overall well-being, sense of stigmatization, and treatment outcome of persons with vitiligo are largely dependent on their social acceptance and this is linked with perception and attitude of this disease in a given population. Therefore, this study assessed the knowledge and attitude of the public towards vitiligo.

**Methods:**

A cross-sectional survey was carried out using a self-reported questionnaire distributed to adults living in Mekelle city, Northern Ethiopia from August to November 2019. Individuals who were 18 to 65 years of age and not suffering from vitiligo were included in the study. A self-administered questionnaire that contains a demographic, knowledge, and attitudes parts was used to collect data. Data were entered using Epi Data® version 3.1 and analyzed using SPSS® version 21.

**Results:**

Of the total 368 subjects, 300 completed the questionnaires giving 81.5% response rate. The mean age was 30 ± 8.3 years and the male-to-female ratio was 1.14 : 1. Friends or families were reported as the most common source of information (70%) about vitiligo. The overall vitiligo knowledge was sufficient in 68.3% of the participants. Higher vitiligo-related knowledge scores were recorded by people older than 30 and below 50, those of secondary school graduated or more, urban-dwellers, persons who had heard about vitiligo, and persons having families or friends affected by vitiligo. Attitudes towards vitiligo were positive in 43.3% of participants. This was more prevalent among employed persons, those of secondary school graduated or more, and persons having families or friends affected by vitiligo. Moreover, sufficient knowledge was significantly related to positive attitudes towards the disease (*p* < 0.0001).

**Conclusion:**

Even though the majority of the respondents had sufficient knowledge, we still found misconceptions and negative attitudes towards vitiligo. Therefore, it is still crucial to educate the public about vitiligo to ultimately improve the well-being of patients with vitiligo.

## 1. Introduction

Vitiligo is a skin depigmentation disease that results from an autoimmune process and is characterized by the destruction of melanocytes [1]. It is estimated to affect 1% of the world's population, regardless of age, sex, and skin color [2]. This chronic disease results in white patches of skin mainly appearing on visible areas of the body, including face and hands, and is rarely accompanied by itching or other somatic symptoms [3]. Most commonly, the disease begins during childhood or young adulthood with the onset of 10 to 30 years but can occur at any age [4].

Pathogenesis is multifactorial and not clearly elucidated and may include mechanisms involving autoimmunity, intrinsic defects of melanocytes, and oxidative stress [5]. Furthermore, up to 30% of patients have a positive family history, and concordance has been noted in monozygotic twins, indicating that a genetic factor is undoubtedly involved [6]. Perhaps due to unclear pathogenesis, individuals with vitiligo are sometimes blamed in some cultures for incurring the disease. In Iran, women with vitiligo have reported that their disease is seen by others as “punishment by God for sins, or at least, moral and spiritual impurity [7].

A considerable number of patients cannot be adequately helped, or therapeutic success lasts only a short time. Moreover, the patient's quality of life deteriorates with lengthening of the disease duration and with the number of visits to the physician [8]. Thus, cosmetically disfiguring appearances lead to serious psychological problems in daily life [9, 10].

The psychological impact of vitiligo varies greatly from person to person, depending on their condition, social and occupational situation, and psychological well-being. Vitiligo is often most obvious in darkly pigmented individuals, in whom the disease can have profound psychological consequences. These effects range from mild embarrassment to a severe loss of self-confidence and social anxiety, especially for those who have lesions on exposed skin [11, 12].

There are many misconceptions related to vitiligo, which vary from one area of the world to another. The common misconceptions are that the disease is contagious, is nontreatable, is related to a specific kind of food/drinks, is a form of leprosy, is always hereditary, and may lead to skin cancers [13]. In Africa, social, religious, and tribal factors play a significant role in stigmatization. The main contributing factor to social stigma is that vitiligo is considered by many as the wrath of the gods upon an individual, nemesis, a curse, mystery, and spiritual attack [14]. Thus, the social discrimination and stigmatization result in significant changes in their lifestyles: from the choice of clothing, use of sunscreen, and cosmetic camouflage of the lesions to the avoidance of social events or outdoor activities [15–17].

Overall, the patient's well-being, quality of life, sense of stigmatization, and treatment outcome are largely dependent on their social acceptance and this is linked with perceptions of this disease in a given population [18]. Therefore, studies aiming to explore the knowledge and attitude of the public towards vitiligo are timely and very critical, particularly in areas where the disease is not well-recognized, which significantly affects the patients' quality of life. As a result, patients would be advantageous as this study assess the knowledge and attitude of the public towards the disease. To the best of our knowledge, no data is available related to public knowledge and attitude towards vitiligo in Ethiopia, in which the current study aimed to generate inferences for the study setting, Mekelle city, Northern Ethiopia.

## 2. Participants and Methods

A cross-sectional survey was carried out using a self-reported questionnaire distributed to adults living in Mekelle city, Northern Ethiopia, from August to November 2019. Individuals who were 18 to 65 years of age and not suffering from vitiligo were included in the study. However, persons who were unable to read the Tigrigna language were excluded from the study.

The sample size was calculated using a single population proportion formula considering 60% [19] anticipated sufficient knowledge, a confidence level of 95%, and 5% margin of error giving a total of 368 samples.

The survey was distributed to a reasonably representative sample of the general population. Two-stage random sampling was employed. In the first stage, five districts were randomly selected representing the middle, southern, northern, eastern, and western parts of Mekelle city. In the second stage, samples were equally selected from the districts using simple random sampling.

A questionnaire was adapted from a study conducted by Al-Shobaili [20]. It was created by the researchers and validated by two dermatologists. It includes questions related to demographic characteristics of the participants and factors associated with knowledge and attitude towards vitiligo patients. The questionnaire was developed in the English language; then it was translated to the local language which is Tigrigna. The aim of the study was clearly explained to all participants. Participants were shown pictures of vitiligo patients in cases of uncertainty of the condition's appearance. Correctly answered questions that elicited knowledge were given a score of 1, whereas incorrect or unanswered/unsure responses were given a score of 0. Total scores were computed using the summation of individual scores and therefore ranged between 0 and 18. The percentage of total knowledge score was computed for each participant and utilized for statistical comparisons. By following the study of Al-Shobaili [20], those who scored at or above the median value were considered as having “sufficient knowledge,” whereas those who scored below the median level were considered as having “insufficient knowledge.”

Questions that explored attitudes towards vitiligo patients were given a score of 2 if positive and 0 if negative. The total score was computed using the summation of individual scores and therefore ranged between 0 and 20. The percentage of the total attitude score was computed for each participant and utilized for statistical comparisons. Those who scored at or above the median value were considered as having a “positive attitude,” whereas those who scored below the median level were considered as having a “negative attitude” [20].

Respondents were asked to fill in the questionnaire in less than 10 min and return to the researcher immediately. Five trained data collectors were recruited for this purpose. A pilot study was conducted on 25 volunteers from different districts to help in the adaptation and modification of the questionnaire. Each respondent was assured that the results of the survey would be used only for research purposes, without any disclosure of identity.

Data were entered using Epi Data® version 3.1 and analyzed using SPSS® version 21. Frequency distributions and percentages were utilized to describe categorical variables, whereas the measure of central tendencies and dispersion were used to describe continuous variables. Independent *T*-test and chi-square tests were applied to test for the association between compared variables. A *p* value at or less than 0.05 was considered statistically significant.

## 3. Results

### 3.1. Demographics

Of the total 368 subjects, 300 completed the questionnaires giving 81.5% response rate. The mean age was 30 ± 8.3 years and ranged between 18 and 65. The gender group was composed of an equivalent number of males and females giving a 1.14 : 1 ratio. Majority (249, 83%) were graduated from secondary school or above. Details regarding demographic characteristics are summarized in Table 1.

### 3.2. Knowledge about Vitiligo

The median knowledge score of the respondents was 10.0. Based on that, the overall knowledge about vitiligo was sufficient in 68.3% of the participants with the majority of the participants gaining their information about vitiligo from friends or family (70%, 210), followed by social media (10%, 30), medical sources (16.3%, 49), and Internet (3.7%, 11).

As illustrated in Table 2, most of the respondents (84.4%) recognized that vitiligo is not contagious by having a meal together, by touching (80.75%), and by air transmission (83.4%). Moreover, 82% of the participants responded that it is more prevalent and exaggerated with exposure to psychological stress. Near to three-fourths of the participants believed that vitiligo is not caused by witchcraft (78.3%), the evil eye (76%), sharing things (73.3%), and evil spirits (72%). Approximately two-thirds of them responded that vitiligo is not an infectious disease (66.3%), not a dangerous disease (66%), not a hereditary disease (65.3%), and not caused due to a sin of God (64%). Above half of them recognized that vitiligo is not caused due to lack of hygiene (54%) and is not leprosy (52%) and near to one-third of them recognized that vitiligo is not associated with the habitual intake of certain foods (36%), caused by unknown etiology (32.3%). Only a few of the respondents recognized other important features of the disease including that it is treatable (16.7%), and it is a disease of the immune system (12.3%).

### 3.3. Factors Associated with Knowledge about Vitiligo

Respondents aged between 31 and 50 years had significantly higher knowledge scores than those aged 18–30 years and >50 years (10.9 ± 2.99 versus 9.5 ± 3.42 versus 8.91 ± 3.27, respectively, *p* < 0.0001). Regarding educational status, secondary school or above graduated respondents had higher knowledge scores compared to primary school graduates (10.55 ± 2.96 versus 7.37 ± 3.73). The difference was statistically significant (*p* < 0.0001). Furthermore, those who reported having heard about vitiligo had a higher significant knowledge score compared with those who had not heard a lot about vitiligo (10.40 ± 3.27 versus 8.92 ± 3.23, *p* < 0.001). Friends or families of persons with vitiligo reported higher knowledge score than who had not (11.48 ± 3.39 versus 9.53 ± 3.16). This also had a statistically significant difference (*p* < 0.0001). However, gender, employment status, and the source of information were not significantly associated with the knowledge score of vitiligo (Table 3).

### 3.4. Attitude towards Vitiligo

As shown in Table 4, most participants would sympathize with patients with vitiligo (79.3%), agreed to continue their marital life (73.3%) if vitiligo happened, and more than half (51%) do not stare at vitiligo patients. The study also revealed that less than half of the respondents would be willing to employ a vitiligo patient as employers (46%), would have the interest to shake hands with vitiligo patients (43.7%), would be interested to share foods with vitiligo patients (39.3%), and would accept food prepared by a vitiligo patient (38.7%). Yet, just above one-quarter (28.3%) would ask vitiligo patients about their disease. Few participants responded that they could date a vitiligo patient (19.3%) and would marry a vitiligo patient (6.7%).

**Figure 1:**
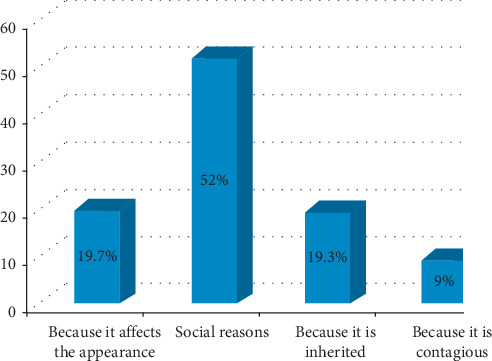
Reasons for refusing marriage among participants (*n* = 300).

The median attitude score of the respondents was 8.0. Thus, the overall attitude towards vitiligo was positive on approximately 43.3% of the participants. As depicted in Figure 1, the main reason to refuse marriage with a person affected by vitiligo was due to social reasons.

### 3.5. Factors Associated with the Attitude towards Vitiligo

As depicted in Table 5, respondents who are graduated from secondary school or above had a higher attitude score compared to primary school graduates (8.83 ± 4.69 versus 6.98 ± 4.35). The difference was statistically significant (*p* < 0.010). In addition to that, employed persons had a higher attitude score than unemployed ones with a statistically significant difference (9.18 ± 4.55 versus 7.65 ± 4.73) (*p* < 0.005). More importantly, friends or families of persons with vitiligo reported a higher attitude score than those who had not (10.81 ± 4.54 versus 7.76 ± 4.49). This also had statistically significant difference (*p* < 0.0001). However, gender, age, residence, information heard about vitiligo, and source of the information were not significantly associated with the attitude towards vitiligo.

### 3.6. Association of Knowledge and Attitude towards Vitiligo

Respondents with sufficient knowledge about vitiligo tended to have significantly higher positive attitudes towards the disease (*p* < 0.0001) (Table 6). Thus, those who were equipped with sufficient knowledge about vitiligo had a 2.8 times higher likelihood of having a positive attitude towards vitiligo compared to persons who had insufficient knowledge.

## 4. Discussion

The focus of the current study was exploring the knowledge and attitude of the general public towards vitiligo. This was found to be essential because studies [21, 22] have documented that the knowledge and attitude of the public tend to affect the lives of many patients with vitiligo.

Surprisingly, the overall knowledge about vitiligo was sufficient in the majorities of our respondents. Thus, nearly two-thirds of the respondents identified vitiligo as a noninfectious disease, not hereditary, and not dangerous. However, Alghamdi and his colleagues reported a higher proportion of (80%) subjects who responded that vitiligo is a noninfectious disease [22]. Moreover, above half of our respondents knew vitiligo as being different from leprosy. However, only a few of them knew that vitiligo is a disease of the immune system and it is treatable. Be that as it may, lack of adequate knowledge regarding the nature of vitiligo might result in unnecessary isolation and discrimination of patients with vitiligo.

Regarding the causes of vitiligo, the current study reported a relatively higher proportion of subjects who responded that vitiligo is not contagious by having a meal together, touching, air transmission, and lack of hygiene. These findings were also supported by Asati and his colleagues [23]. Besides, the majority of the respondents recognized that vitiligo is more prevalent and exaggerated with exposure to psychological stress. However, certain misconceptions which were commonly reported are as follows: vitiligo is caused by certain foods, witchcraft, evil eye, and evil spirit. These findings were also reported by a study done in western Saudi Arabia [19]. Thus, education on the causes of vitiligo might enable the public to promptly seek healthcare.

Higher vitiligo-related knowledge scores were recorded by people older than 30 and below 50, those graduated from secondary school or above, urban-dwellers, persons who heard about vitiligo, and persons having families or friends affected by vitiligo. This was in line with findings reported by Alghamdi and his colleagues [22]. Therefore, as revealed by Kent and Al'Abadie [18], misconceptions related to vitiligo might affect lives in a variety of ways, mainly due to activities such as avoidance and negative reactions by others. Thus, it might be important to seek healthcare early if someone, from the public, is suddenly affected by vitiligo.

In our study, the most commonly reported positive attitudes towards vitiligo were sympathizing for patients having vitiligo, continuing marital life if vitiligo happens in between, and not staring at vitiligo patients. However, around half of the respondents would not be willing to employ a vitiligo patient due to fearing of losing their customers, would have no interest to shake hands with vitiligo patients, had no interest to share foods with vitiligo patients, would not accept food prepared by a vitiligo patient, and would not ask vitiligo patients about their disease for the safety of the individuals affected by vitiligo. The worst response was that most of the respondents do not want to date someone affected by the disease and would not be willing to marry individuals who are affected by vitiligo. Though this was responded by both sexes, it was more common among males who are single due to social reasons. This might be the reason for the common difficulties that vitiligo patients, particularly single women, experience when attempting to initiate relationships [24, 25].

The positive attitudes towards vitiligo were more prevalent among employed persons, those graduated from secondary school or above, and persons having families or friends affected by vitiligo. This had an agreement with some previous studies [19, 26]. Therefore, education about vitiligo targeting especially those who are unemployed, lower grade educated persons, and anyone who has no families or friends affected by the disease might bring change in attitude that could prevent the isolation of patients with the disease.

In the present study, we found that the public's knowledge about vitiligo had a significant relationship with the attitude towards vitiligo. Thus, participants with sufficient knowledge about vitiligo tended to have more positive attitudes towards the disease. This concurs with previous studies by Juntongjin et al. [27] and Fatani et al. [19]. Therefore, awareness creation about the disease is believed to be a core step to change the attitude of the public towards the disease.

## 5. Conclusion

Even though the majority of the respondents had sufficient knowledge, we still found misconceptions and negative attitudes towards vitiligo. Besides, uncommon misconceptions were also noted including the belief that vitiligo is caused by witchcraft, evil eye, and evil spirit. A considerably high number of respondents also reported that they will not marry a person with vitiligo. Therefore, educating the public about vitiligo is crucial to increase self-confidence, social integration, and psychological well-being of patients with vitiligo.

### 5.1. Limitation of the Study

The limitations of this study were the small sample size and the potential for the introduction of biases associated with self-reporting and under or overreporting of information.

## Figures and Tables

**Table 1:** Demographic characteristics of the participants (*n* = 300).

Variables		Frequency	Percent
Gender	Male	160	53.3
	Female	140	46.7
Age (mean ± SD)	30 ± 8.3		

Age group	18–30	141	47
	31–50	122	40.7
	>50	37	12.3

Marital status	Single	137	45.7
	Married	147	49
	Divorced	13	4.3
	Window	3	1

Educational status	Primary	51	17
	Secondary or above	249	83

Occupation	Employed	170	56.7
	Unemployed	130	43.3

Residence	Urban	283	94.3
	Rural	17	5.7

SD = standard deviation.

**Table 2:** Response of the participants to knowledge questions about vitiligo (*n* = 300).

Questions	Correct answers	
	*N*	%
Vitiligo is more prevalent and exaggerated with exposure to psychological stress “YES”	246	82
Vitiligo is a disease of the immune system “YES”	37	12.3
Vitiligo caused by unknown etiology “YES”	97	32.3
There is a treatment for vitiligo “YES”	50	16.7
Vitiligo is a hereditary disease “NO”	196	65.3
Vitiligo is an infectious disease “NO”	199	66.3
Vitiligo is associated with the habitual intake of certain foods “NO”	110	36.7
Vitiligo contagious by sharing things “NO”	220	73.3
Vitiligo caused by lack of hygiene “NO”	162	54
Vitiligo caused by evil eye “NO”	228	76
Vitiligo caused by witchcraft “NO”	235	78.3
Vitiligo caused by evil spirit “NO”	216	72
Vitiligo caused due to a sin of God “NO”	192	64
Vitiligo is leprosy “NO”	156	52
Vitiligo contagious by touching “NO”	242	80.7
Vitiligo contagious by having a meal together “NO”	254	84.7
Vitiligo contagious by air transmission “NO”	250	83.4
Vitiligo is a dangerous disease “NO”	195	66

**Table 3:** Factors associated with the knowledge about vitiligo (*n* = 300).

Variable		Vitiligo knowledge score percentage		*p* value
		Mean	SD	
Gender	Male	10.06	3.28	0.826
	Female	9.97	3.39	

Age	18–30	9.50	3.42	0.0001^*∗*^
	31–50	10.94	2.99	
	>50	8.91	3.27	

Educational level	Primary	7.37	3.73	0.0001^*∗*^
	Secondary and above	10.55	2.96	

Employment	Employed	10.33	3.50	0.053
	Unemployed	9.60	3.03	

Residence	Urban	9.96	3.34	0.020^*∗*^
	Rural	10.94	2.96	

Heard about vitiligo	Yes	10.40	3.27	0.001^*∗*^
	No	8.92	3.23	

Source of information	Friends or family	10.15	3.17	0.678
	Social media	9.63	3.05	
	Medical source	9.61	4.04	
	Internet	10.18	3.42	

Friends or families affected by vitiligo	Yes	11.48	3.39	0.0001^*∗*^
	No	9.53	3.16	

^*∗*^Statistically significant: *p* ≤ 0.05; SD = standard deviation.

**Table 4:** Response of the participants to attitude questions about vitiligo (*n* = 300).

Questions	Participant's response	
	Yes *n* (%)	No *n* (%)
I would sympathize for a patient having vitiligo	238 (79.3)	62 (20.7)
I would ask vitiligo patients about their disease	85 (28.3)	215 (71.7)
I stare patients with vitiligo	147 (49)	153 (51)
I would avoid shaking hands with a vitiligo patient	131 (43.7)	169 (57.3)
I would eat food prepared by a vitiligo patient	116 (38.7)	184 (61.3)
I would share food with vitiligo patient	118 (39.3)	182 (60.7)
As an employer, I would hire a vitiligo patient	138 (46)	162 (54)
I would dating	58 (19.3)	242 (80.7)
I would marry a vitiligo patient	20 (6.7)	280 (93.3)
I would continuing marital live with vitiligo patient	220 (73.3)	80 (26.7)

**Table 5:** Factors associated with the attitude towards vitiligo (*n* = 300).

Variable		Vitiligo knowledge score percentage		*p* value
		Mean	SD	
Gender	Male	8.65	4.65	0.590
	Female	8.36	4.73	

Age	18–30	8.52	5.00	0.664
	31–50	8.69	4.49	
	>50	7.89	4.11	

Educational level	Primary	6.98	4.35	0.010^*∗*^
	Secondary and above	8.83	4.69	

Employment	Employed	9.18	4.55	0.005^*∗*^
	Unemployed	7.65	4.73	

Residence	Urban	8.47	4.68	0.572
	Rural	9.18	4.90	

Heard about vitiligo	Yes	8.65	4.72	0.394
	No	8.12	4.58	

Source of information	Friends or family	8.18	4.48	0.161
	Social media	8.40	5.21	
	Medical source	9.75	5.16	
	Internet	9.63	4.27	

Friends or families affected by vitiligo	Yes	10.81	4.54	0.0001^*∗*^
	No	7.76	4.49	

^*∗*^Statistically significant: *p* ≤ 0.05; SD = standard deviation.

**Table 6:** Association between attitude and knowledge regarding vitiligo among participants.

		Attitude towards vitiligo		*p* value
		Negative (*N*, %)	Positive (*N*, %)	
Knowledge about vitiligo	Insufficient	70 (73.7)	25 (26.3)	0.0001^*∗*^
	Sufficient	101 (49.3)	104 (50.7)	

^*∗*^Statistically significant: *p* ≤ 0.05.

## Data Availability

The data used to support the findings of this study are included within the article.
